# Hospital and Institutional News

**Published:** 1917-06-30

**Authors:** 


### HOSPITAL AND INSTITUTIONAL NEWS.

### THE MESOPOTAMIA REPORT.

THE report which has just been issued by the Commission appointed in August 1916 to inquire into the causes of failure of the Mesopotamia Expedition deserves the close study of all those who wish to know the causes of the unfortunate mistakes of the past and who hope that similar errors may be avoided in future. The report as a whole appears to us to be fearless and fair: blame has been laid on those who seem to the Commissioners to be deserving of it, and praise is given where it seems to be merited. With the medical portion of the report we are chiefly concerned, and a perusal of it gives rise to a feeling of regret that the medical work was carried out so inefficiently. With the individual officers who are responsible for the failures we are not immediately concerned. Faults indeed there were, and great faults; not only are individuals responsible, but the whole system was unequal for coping with warfare on the scale in which the war in Mesopotamia was waged. The extensive system of check and countercheck existing in the management of the Indian Medical Department may perhaps have been well enough for times of peace, or even for a small frontier war, but it was utterly unsuited for dealing with the medical work of an expedition such as that which endeavoured to make its way up the Tigris banks. When the medical officer in charge of the arrangements for such an expedition cannot "off his own bat" order the equipment and stores in his opinion needed by it, but his requisitions have to be passed and approved by several officials who have no expert knowledge of the matter, not only is valuable time lost but there is no little risk that reductions will be made in order to save a little money at the cost of efficiency in one of the most important branches of a modern army.

### THE SYSTEM AT FAULT.

THE beginning of the evil antedated even the beginning of the war. The neglect of the Army in this country had extended to India. There was a shortage in those stores and materials even below the amount which had been considered sufficient for times of peace. Can it, then, be a matter of wonder that when the strain of the preparation for what was really a great expedition came that the medical department was found ill-equipped for the work it had to do? The number of medical officers fell short of what were needed. Only twelve sections of field ambulance were supplied for each division, although twenty should have been furnished. There was not a single hospital river steamer, though everyone knew that the river Tigris would figure largely as a channel of transit. Yet the sick and wounded numbered thousands. They had to be carried by the ordinary transport vessels already over-burdened by the ordinary river transport work. These boats were in some cases used without undergoing any cleansing after they had been employed for carrying animals. Not a single wheeled ambulance wagon was provided, so that the wounded had to endure unnecessary shaking and suffer much unnecessary pain. The report makes sad reading for all those who feel how much should be done for our wounded, and especially is it painful for those to read who had fathers, brothers, or sons among the wounded. If the campaign had been carried on in a land with a temperate climate the suffering would have been great, but it was carried on in a climate surpassed by few in this world for heat and dust. The very commonest needs of the sick and wounded were often not supplied, and all this pain and suffering could have been prevented by care and forethought. Some of the officers were certainly to blame; and some, notably Major Carter, by his exhibition of plain speaking and courage, deserve the thanks of the nation. But, in addition to this and beyond this, is the fault of the system, which does not give a free hand to those who are really responsible for the carrying out of such arduous duties as fell to the lot of the medical department of the Mesopotamia Expedition.

### VICE-CHANCELLOR SIR COOPER PERRY, M.D.

THE London University is to be congratulated upon securing as its Vice-Chancellor for the year 1917-18, in succession to Sir Alfred Gould F.R.C.S., Sir Cooper Perry, M.D., F.R.C.P., who is widely recognised as one of the ablest administrators, with probably the most knowledgeable, cultivated, and capable brain to be met with in the medical profession. Sir Cooper is a son of the late Rev. E. Cresswell Perry, Vicar of Seighford, Staffordshire. He was educated at Eton and King's College, Cambridge, and won the highest attainable position in the schools. He possesses great musical talent, as King's College chapel and its services demonstrate. Sir Cooper is a consulting physician, and physician to the skin department of Guy's Hospital, of which he is the superintendent. He has done useful work as an examiner at Cambridge and London University, and has rendered valuable service to the hospitals as an active member of the Distribution Committee and General Council of King Edward's Hospital Fund for London. He has taken an active part in the affairs of the Evelina Hospital for Children, Southwark, and has done yeoman service in many other ways in the district surrounding Guy's Hospital. Sir Cooper possesses untiring energy. Everything he undertakes is done in a masterly and thorough manner, and there are few, if any, more valuable workers than himself in the fields of work which have had the good fortune to secure his interest and personal service. Sir Cooper married in 1890 the daughter of Mr. James McManus, of Killeaden House, Co. Mayo. Lady Perry is deservedly popular. She has the power of organisation and accomplishment, and has done much useful work in many directions. The home atmosphere of the former treasurer's house at Guy's, where Sir Cooper and Lady Perry reside, has a charm which gives refreshment to all who share the privilege of their friendship.

### THE UNITED STATES RED CROSS.

THE United States Red Cross has dispatched a mission consisting of nine members to France to investigate the immediate relief needs. It will make preparations for the coming of the United States troops, and will remain in Europe during the rest of the war. It will report to the Red Cross War Council in Washington. When the Commission arrives in France it will appoint a representative in every district of France where it seems to be necessary, and its activities will extend to other countries if needed. Major Grayson Murray, who is in charge of the mission, is by profession a banker, and some of the other commissioners are also bankers; the object of choosing such commissioners is to have the work of relief carried on in a thoroughly businesslike manner. Dr. Alexander Lambert, who is the medical expert on the Commission, intends to study the tuberculosis problem in France. Mr. George B. Ford, another commissioner, visited France recently as a member of the American Industrial Commission, and he intends to consult with the French authorities on the questions involved in the rebuilding of the towns laid waste during the war. It is hoped that much help in this respect will be obtained from America; it will probably take the form of money loans and building materials.

### HOSPITAL IMPROVEMENTS AT THE FRONT.

IN a recent interview Lieutenant-Colonel Gilbert Barling, C.B., who went to France last autumn as consulting surgeon to the base hospitals at Rouen, emphasised two improvements in the treatment of wounded at the Front. The first concerns the more rapid treatment of the wounded, which has been now secured by the establishment of a sufficient number of casualty clearing stations five or ten miles behind the fighting-line. Each can deal with about 1,000 men, and thus the old and sometimes disastrous delay caused by sending the men after first-aid treatment on a long train journey to the base hospitals has been avoided. He points out also the immense advantage of having a special hospital for abdominal cases in the same forward position. The second improvement concerns the cleansing of wounds, and we are all familiar with the controversies which different methods have excited. Colonel Barling praised the Carrel system, which originated in America, and consists in flooding a wound every two hours with an antiseptic. Since the wounds caused by high-explosive shells are always septic, it is easy to understand the various attempts to deal with them, and the friendly rivalry of different methods.

### LEICESTER FARMERS' AND GRAZIERS' SPONTANEOUS GENEROSITY.

AN interesting ceremony took place at the Leicester Royal Infirmary on Wednesday, when a bed, endowed by farmers and graziers, was formally unveiled. Mr. Fred Horton, who took a great interest in the organisation for the collection of funds, in handing to Mr. Fielding Johnson, jun. (the chairman of the infirmary governors), a cheque for £1,000, congratulated the farmers and graziers of the district in being "first in the field" to have a bed endowed by their collective contributions. He mentioned that the idea owes its origin to an anonymous farmer and grazier, who promised £5 5s. per annum for two years if others would contribute what they could for a like period. The challenge was readily taken up, and well within the period named the required amount was raised. Looking round the "George Oliver" Ward, Mr. Horton observed that there were still other beds to be similarly endowed, and he hoped the farmers would not rest content, but would still further combine in support of the infirmary, for then more endowed beds would speedily be supplied. The tablet was then unveiled by Mr. W. T. Hayr, a well-known local farmer and grazier, and Mr. Harry Johnson, responding to a vote of thanks for his help, expressed the hope that at any rate an effort would be made to endow a cot in the Children's Hospital, a suggestion which was readily taken up, and enthusiasm for it was aroused by an inspection of the Leicester Infirmary which followed the ceremony. It was subsequently announced that women farmers and the wives of farmers and graziers would make themselves responsible for the collection of the required amount (£500) for the children's cot.

### AN EARL KITCHENER MEMORIAL.

FIVE days later the ceremony of unveiling the local memorial to Earl Kitchener, in the shape of an endowed bed in the institution, was performed by Mrs. Parker, Earl Kitchener's sister. A cheque for £1,000, raised by contributions from all sections of the community, was presented to Mr. Fielding Johnson (the chairman) by Miss Phyllis A. Browne, who proved herself the most successful collector, her list totalling £101. The Chairman, in inviting Mrs. Parker to unveil the tablet, referred to Earl Kitchener's great national services and to his local associations with the county through his parental residence at Cossington Hall and Mrs. Parker and her husband's residence at Rothley, within a few miles of Leicester. After the Bishop of Leicester had offered dedicatory prayers, Mrs. Parker unveiled the memorial, which bore the inscription: "This Bed was Endowed by Residents of the Town and County of Leicester as a Memorial to the great National Services rendered by Field-Marshal Earl Kitchener. 1917."

### A HUGE WAR CHARITY WOUND UP.

THE National Committee for Relief in Belgium publicly announced on May 19 last that, as the United States Government had assumed financial responsibility for the cost of relief, appeals to the British Empire would be suspended, and that after June 1, the date when the first payments of the American loan became available, any donations received would "be held to provide for emergencies now unforeseen in connection with relief in Belgium." It will be remembered that the Committee in this country confined its energies to appeal, the expenditure of the money raised being left to a larger and international body. The final report of the Appeal Committee is issued for the period from April 27, 1916, to May 31, 1917. The total collected since the first appeal was issued in 1915 was £2,411,222, but to this must be added sums approximating £100,000, which were sent direct to the parent body by branch funds. A quarter of a million is a respectable contribution from this country and its Dominions and Colonies, especially when it is remembered that there were many other concurrent appeals. But, whatever the sum, it was accompanied by great sympathy with those who are suffering through no fault of their own.

### A NEW HOSPITAL JOURNAL.

The exhibition of work done by wounded and disabled men, which was opened by the Duke of Connaught at Messrs. Sotheby's New Galleries, 34 and 35 New Bond Street, is only the latest indication of the work now being done by wounded soldiers and the endeavour being made to help them back to a useful place in civil life. With this object a handsome new journal, entitled *Recalled to Life*, has been started, and is to be published periodically. It is to devote itself to the "care, re-education, and return to civil life of disabled sailors and soldiers," for whose guidance it has been founded. Sir Alfred Keogh and Sir Robert Jones contribute two important articles on the "Treatment of the Disabled" and "Orthopædic Surgery in its Relation to the War." Every side of the work now being undertaken for the disabled man is reviewed, but the first criticism which suggests itself is that, as at present edited, it is too big, not to say too expensive, for its professed purpose. People who wish to take an intelligent interest in the subject and are prepared to pay a good price will read it, we hope, with avidity; but such a "review" is not the type of paper which disabled men and their relations are likely to turn to for information and assistance in their new professions. The new journal, it is understood, is edited by Lord Charnwood, and the publication is due to the joint efforts of the War Office, the Ministry of Pensions, and the Red Cross, who hope by its means to keep the hospitals, the Pensions Committees, and the education authorities in touch with what is being done. For this purpose a price of two shillings may not be extravagant, but if the disabled sailor or soldier is thought worthy to be placed in possession of this information, it is obviously beyond his reach.

### THE SATURDAY FUND IN BIRMINGHAM.

The collection on behalf of the Birmingham Hospital Saturday Fund is making good progress, and as we write a total of some £30,000 has been achieved, which is an increase of over £4,000 on the sum raised at the corresponding period of last year. The number of contributors has risen also, and we hope that these two facts will bear fruit not only immediately, but in the important time which lies ahead, when the demobilisation of industry and of the Army will make it urgently necessary to see that the Saturday Fund movement generally maintains the lead which it gained under the peculiar circumstances of the war. It is for this reason that the present condition of the Saturday Fund in every centre is worthy of study and attention.

### A WOMAN MEDICAL OFFICER OF HEALTH.

The war has given a great opportunity to medical women to show their fitness for much medical work for which many people did not consider them suited. Very many of them have served at home as resident medical officers in London and in all parts of the country. In the earlier stages of the war hospitals entirely officered by women proceeded to France and gained great credit for the work they did. In Serbia also units staffed by women did good work. Some forty medical women are holding resident posts in Malta in the many hospitals which have been established there. From every one of the scenes of their activities has come an almost unanimous appreciation of their work, and there cannot be the slightest doubt that the calling in of medical women has been of the utmost assistance to the medical profession of this country in the enormous strain that has been placed upon it. There has just been reported a further extension of this principle, for a medical woman has been appointed a medical officer of health. At a recent meeting of the Holland County Council at Boston, Mrs. Marjorie A. Harcourt, M.A., M.B., of London, was appointed medical officer of health and school medical officer for the "Parts of Holland" (a district of Lincolnshire), at a salary of £450 a year.

### DENTISTRY FOR SAILORS AND SOLDIERS.

Soon after the outbreak of the war Miss Fletcher started the Soldiers' and Sailors' Dental Aid Fund, of which she became honorary secretary. She had learned that it was not possible for sailors or soldiers to obtain artificial teeth unless they or their friends paid for them, and she started the fund to supply this deficiency. In November 1915 the War Office and the Admiralty took over the provision of teeth for sailors and soldiers on active service, but the Fund continued to provide these necessities for the Home Army and the Merchant Service. After being closed for four months it was reopened and received the name of the "Ivory Cross," or the National Dental Aid Fund, and it is now carrying on its beneficent work for men discharged from the Services, for the Home Army, the Merchant Service, and the necessitous poor, amongst whom the women and children receive the benefits of the Fund as well as the men. General Sir Francis Lloyd, who commands the London District, expressed his approval of the scheme, and explained that, although the War Office provided artificial teeth, if there were need, for men who were going into the firing line, yet men who were behind the firing line, even though they were in khaki, were unfortunately not supplied with artificial teeth. The Fund deserves support, for the provision of suitable and satisfactory artificial teeth is generally utterly beyond the means of the poor, and as health depends so greatly on the perfection of the teeth it is distinctly for the public good that the poor should be provided with good teeth. The Insurance Act, from which some expected so much, has never attempted to do anything for the teeth of the poor.

### THE PAY OF TERRITORIAL MEDICAL OFFICERS.

When war broke out in 1914 Territorial medical officers were called up, and they had to abandon their civil work at almost a moment's notice. In some cases it was possible to obtain a *locum tenens* who for a time kept the practice together, but in many instances even this was not possible, and the practice ceased to exist. The work which these Territorial officers have to perform is in many cases by no means small, and yet we are told the pay even of one who has attained to the rank of a captain is only fifteen shillings and sixpence a day. With this may be contrasted the pay of a lieutenant of the Royal Army Medical Corps, which commences at twenty-four shillings a day. The difference is certainly striking, and we can see no possible explanation of the distinction. It does not connote any difference of professional standing or real difference in the amount of work done, and unless there is some explanation hidden from us it is a matter which should have the earnest attention of those in authority. The fact that the Territorial medical officer was, during peace time, willing to undertake the somewhat thankless task of doing Territorial work hardly appears to be a valid reason for depriving him of emoluments freely given to his brother practitioner who for one reason or another did not do any Territorial work in times of peace.

### FOUNDER'S DAY AT ALTON CRIPPLES' HOSPITAL.

"Founder's Day" at Lord Mayor Treloar Cripples' Hospital, Alton, Hants, always an interesting function, was this year of special interest, owing to the fact that, coinciding with the anniversary of the Battle of Jutland, it was made the occasion for the dedication of a special memorial ward, founded in memory of the officers and men of the 4th Destroyer Flotilla who lost their lives in that battle. The Lord Bishop of Guildford conducted the service of dedication, assisted by the Rev. C. R. S. Elvin, chaplain at the hospital. The Rev. F. G. G. Jellicoe, brother of Sir John Jellicoe, was present, and amongst the guests were Rear-Admiral Halsey (Third Sea Lord) and Fleet-Surgeon Hewitt, representing the Admiralty. Responding to the toast of "His Majesty's Forces" at lunch, Rear-Admiral Halsey said that Sir Edward Carson had requested him to express his deep sympathy with the great work that was being done at Alton. Sir John Jellicoe would have been there if he could have spared the time from his duties, for he took great interest in the hospital and in the foundation of the memorial ward. Sir William Treloar announced the receipt of a message from Queen Alexandra expressing warm appreciation of the tribute paid to the brave seamen who had given their lives for King and country, and wishing the scheme every success.

### A FLOATING HOSPITAL.

A number of medical men lately inspected the new hospital ship which has been built for work on the Tigris by Messrs. Livingstone and Cooper, of Hull, for the Inland Waterways and Docks Department of the War Office. Its chief points of interest are its small draught, its exceptionally powerful steering gear, and its system of artificial ventilation. The Tigris is a river with many shallows and very sharp turns, and for these reasons the new boat draws only 3 feet 6 inches, and is provided with four rudders. With a length of 160 feet and four decks it can accommodate some 200 cases, and has a speed of ten miles an hour. Apart from the electric fan which circulates the air to every group of four beds, a continuous supply of cool or warm air is provided by a ventilation system, since the winters are as cold as the summers are hot. Artificial systems of ventilation have not permanently established themselves so far in relation to buildings on land, but we can well believe that another attempt in their direction is essential for hospital ships intended to ply in Mesopotamia. The ship is a complete unit, with its own operating theatre and convalescent deck. Its equipment includes an aerating soda-water machine, and, of course, a refrigerating plant.

### THE EAST PRESTON GUARDIANS AND THEIR INFIRMARY.

Though several workhouses have been taken over by the military in the district, the East Preston Guardians are expecting that their entire infirmary will be requisitioned, and added to the list of Sussex infirmaries already occupied by the wounded. Here, as elsewhere, the basis of the change will be that no profit and no loss should accrue to the Guardians, who have agreed, after some discussion, to accept the proposal when it is formally made, and to empower the chairman and clerk to deal with matters of urgency.

### A COTTAGE HOSPITAL PLAN OF CAMPAIGN.

The Woolwich and Plumstead Cottage Hospital Association has decided to postpone a scheme for the extension of its present site and buildings, which has not been able to secure official encouragement. But on the other hand careful plans are being made to collect the increasing funds so that immediate advantage may be taken of the earliest opportunity. Four plans, which may be simultaneously carried on, have been forwarded. The first and chief is the organisation of a weekly collection in the workshops of the district, the sum being left to the discretion of the separate organisers. The second is for the holding of a big bazaar in the autumn, which it is hoped that perhaps Princess Mary may be persuaded to open. The two final plans are for the holding of a Flag Day, and for an advertisement campaign in the local papers. This last, of course, would be in one sense automatically secured if the three former efforts were successful; since to make a hospital the centre of public activity is to fill men's thoughts with its needs, which as topics of the hour are sure to be chronicled sympathetically and at length in the newspapers of the district.

### THE SCARCITY OF DOCTORS.

THAT the Army must have more doctors appears to be certain, but without cavilling in any way at the decision of the medical department of the Army we may be allowed to point out that the civil population is being deprived in some parts of the United Kingdom of the medical aid that it needs. At a recent meeting of the Lancashire Insurance Committee an inquiry was made as to what steps were being taken to remedy the great reduction of the medical men in the district in consequence of so many having been called up. It was stated that 241 panel doctors in the county had been called up, and the number of their panel patients was nearly 164,000. It was stated in reply that the panel doctors who had not been called up had arranged to look after the panel patients of those doctors who had received commissions in the Royal Army Medical Corps. We hope that due attention is being paid to this point in other districts, for if not there will be much unnecessary suffering amongst patients on the panel.

### THE HOSPITAL TREATMENT OF VENEREAL DISEASES.

THE clinics for the treatment of venereal diseases which have been established in more than twenty hospitals in London and its suburbs have now been working for more than five months, and so far most of them have had a large and, what is more important, an increasing, number of patients. Now it is thought desirable that a knowledge of the existence of these free clinics should be more widespread, so the public health authorities are sending out to every householder a circular giving particulars of the clinics with the times at which patients can be seen. The circular lays stress on the importance of the early and efficient treatment of these diseases, and urges that all suffering from them should apply for treatment without delay. Many of the clinics are held in the evening for the convenience of the working classes; the treatment is absolutely free, and in order to make attendance easy for those who do not wish to be known each patient is given a number, which is attached to his prescription paper, and his name is not employed.

### EDUCATION AND PUBLIC HEALTH.

THE intimate correlation between the physical condition of the rising generation and their progress in school studies is, it seems, at last being recognised by the educational authorities. "It is not enough," says the Annual Report of the Board of Education, "to remedy defects; it is essential also to take steps to improve the general physique as well. The number of rejections from the Army on grounds of physical unfitness has emphasised the need for action." In February 1917 the Board were able to announce that funds had been placed at their disposal to enable them to pay grants in aid of expenditure to local authorities for the employment of competent persons to organise and supervise the physical training of children in public elementary schools. It is hoped that these grants will lead to a great improvement in the organisation and methods of the physical training which is given in the public elementary schools, and thereby to an improvement in the health and physical efficiency of the children.

### THE VOTING SYSTEM IN ABEYANCE.

A DECISION come to by the committee of the Royal Alfred Aged Merchant Seamen's Institution in respect of the usual election will, we are confident, meet with universal approval. In view of the shortage of labour and the national need for economy, the election which would have been held this month will not take place, and the voting papers will not be issued. The committee will fill from the waiting list the vacancies at the home on the rising ground by the river Thames at Erith and grant out-pensions to other eligible and approved candidates on the list. The Royal Alfred Institution is now completing its first half-century of service on behalf of British seamen, and there never was a time when greater sympathy could be elicited in a practical form for this and other charities of good standing who look after the welfare of the humble representatives of the Mercantile Marine. It will probably be found by the committee that the relaxation of the voting system, which so often bears hardly upon the claims of the individual and which never satisfactorily ensures the placing of charitable benefits in exactly the right quarter, will by no means have the effect of decreasing public financial support.

### INFANT WELFARE IN LIVERPOOL.

AN interesting statement was made at the last meeting of the Liverpool Corporation showing what the city is doing in regard to infant welfare. The scheme which is now being worked is divided into three parts—ante-natal, natal, and post-natal. Ten centres have been provided for expectant mothers, at each of which a doctor and a trained nurse are in attendance, and free medical advice is given. In addition, the Maternity Hospital has opened in Chatham Street a rest home, where women are treated. Home visiting is carried out by the midwives of the city, and these experienced women are followed by the female health visitors. Complicated cases are dealt with at the Maternity Hospital (twenty beds), at the rest home (twelve beds), and at several of the city hospitals; but, as Liverpool has a birth-rate of over 20,000 per annum, this accommodation is totally inadequate, and negotiations are proceeding with the Maternity Hospital Committee for ampler provision. The doctors' fees at confinements are paid, half being refunded by the Local Government Board. Thirteen schools for mothers—for the teaching of cooking, knitting, and sewing—are being opened, and girls from the elementary schools receive instruction in mothercraft and hygiene. Shortly the day nurseries will be eight in number, and forty beds are available at Leasowe for children suffering from wasting diseases. Close supervision is given by practical nurses in the matter of ophthalmia neonatorum, and no cases of blindness from this cause have occurred in recent years. As circumstances permit, the Infant Welfare Sub-Committee intends to extend further its operations by increasing the number of the agencies brought into the scheme and to strengthen the services mentioned above.

### WHAT THE NURSE HAS WON FOR WOMEN.

THE establishment of the Order of the British Empire and that of the Companions of Honour, which are open equally to women and to men, is one which many will regard as more significant than the acceptance of the principle of woman suffrage by Parliament. The latter may have been bound to come. The former would not have been in sight had not the war proved the services of women to be indispensable. Women who become Companions of Honour receive neither title nor precedence. Dames of the Order of the British Empire have titles, but take precedence after those who possess titles through inheritance or marriage. While research reveals that women have been admitted into various Orders in the past, and, indeed, are admitted to-day into certain home and foreign Orders, most people will regard the bestowal of the Order of Merit on Florence Nightingale as the first step in modern times towards the new departure. It is noteworthy that women of the present day have gained titular distinction mainly through their work for the sick. It was for this Miss Nightingale received the Order of Merit; it was for this that women were admitted to the Order of Mercy and the Order of St. John of Jerusalem. Those women who have received the Kaisar-i-Hind Medal have often had a similar claim, and the Royal Red Cross speaks for itself. The profession of nursing thus may claim to have been the chief cause of winning this public recognition of women's work. This happy result occurs, too, at a time when the profession is setting its own affairs in order through the creation of the College of Nursing.

### CHARWOMEN'S PIN-MONEY.

THE appointment of a small committee to inquire into the working conditions of the women who are given charing work at Brownlow Hill Infirmary in return for relief has led to a curious discussion. It was stated that some of these women regarded the relief which they received as pin-money, since they were receiving allowances from their male relations in the Army. This attitude is disliked by the Guardians. Surely either the allowances so received are sufficient to maintain the women, in which case any relief they may receive is, in fact, an extra, or without the relief they cannot maintain themselves. If they can afford to be independent, the Guardians must adopt some other plan.

### A PROSPEROUS YEAR.

THANKS to the combined results of special subscriptions, receipts from the War Office, and the generosity of the Saturday and Sunday Committee, the Beckett Hospital, Barnsley, has closed the year with a handsome balance of £800 in hand. It is also receiving the endowments of several beds in memory of fallen soldiers, and doubtless this number will be added to before long. Colonel T. W. H. Mitchell is commemorating the death of his son by endowing a bed, the local movement for the endowment of a bed in memory of men from the district who have been killed is approaching success, while the Miners' Federation of Great Britain is about to endow a bed in memory of the late Mr. Pickard, M.P., of Barnsley. It is encouraging to read such signs of prosperity at the present time, especially when this prosperity is not such a piece of good fortune as a legacy may provide, but the result of widespread local interest in many directions.

### THE END OF A SERIES.

THE summer number of the *Craigleith Hospital Chronicle* is the last but one to appear monthly. The monthly series will cease with volume v., which comprises the first thirty issues, and even this plan has had to be modified. "Owing to unavoidable circumstances, the concluding number of the volume will not appear in July, but is being postponed till September. Since the *Chronicle* has maintained the high standard of contents and appearance and paper with which it began, there is bound to be disappointment. The editor is still able to rely upon a staff of writers who are not connected with the institution, and the result is that the contributions cover a wide ground, and have a professional flavour about them which is often not found in hospital gazettes. History, sketches, verse, and a "letter from Baluchistan" indicate its scope, and in appearance it stands alone for excellence so far as our experience goes.

### THIS WEEK'S DRUG MARKET.

OWING to the steady rise in prices and the difficulty of obtaining supplies, business is becoming increasingly difficult. Camphor is unchanged since last week's advance, but makers are disinclined to accept new orders as their commitments forward are so heavy. Remarkable prices are asked for saccharin. A small business continues to be done in quinine, and the price is practically unchanged. The advanced prices of salicylic acid and sodium salicylate are firmly maintained, and, in view of the inadequacy of supplies, a further advance is by no means improbable. There is no change in the position of bromides. Business is reported in balsam Peru at a high figure. Cream of tartar is again rather dearer. Phenacetin has become still scarcer and the price is advancing. Cassia oil is dearer. Epsom salts has advanced in price. The Ministry of Munitions has fixed a maximum price for castor oil.

### TO OUR READERS.

CONTRIBUTIONS are specially invited from any of our readers to these columns. They should deal with topical subjects and news. They must be authenticated for the information of the Editor only. The minimum payment if published is 5s. There is no hard-and-fast rule as to space, but notes of about twenty lines in length are preferred.

